# Lead Screening for HIV of C-C Chemokine Receptor Type 5 Receptor Inhibited by Traditional Chinese Medicine

**DOI:** 10.1155/2014/313094

**Published:** 2014-04-30

**Authors:** Tzu-Chieh Hung, Kuen-Bao Chen, Hung-Jin Huang, Calvin Yu-Chian Chen

**Affiliations:** ^1^Department of Biomedical Informatics, Asia University, Taichung 41354, Taiwan; ^2^School of Medicine, College of Medicine, China Medical University, Taichung 40402, Taiwan; ^3^Department of Anesthesiology, China Medical University Hospital, Taichung 40447, Taiwan; ^4^Department of Chinese Pharmaceutical Sciences and Chinese Medicine Resources, College of Pharmacy, China Medical University, Taichung 40402, Taiwan

## Abstract

The acquired immunodeficiency syndrome (AIDS), caused by the human immunodeficiency virus (HIV), has become a serious world-wide problem because of this disease's rapid propagation and incurability. Recent research has pointed out that the C-C chemokine receptor type 5 (CCR5) is an important target for HIV infection. The traditional Chinese medicine (TCM) database (http://tcm.cmu.edu.tw/) has been screened for molecular compounds that, by simulating molecular docking and molecular dynamics, may protect CCR5 against HIV. Saussureamine C, 5-hydroxy-L-tryptophan, and abrine are selected based on the docking score being higher than Maraviroc and other TCM compounds. The molecular dynamics are helpful in the analysis and detection of protein-ligand interactions. According to the docking poses, hydrophobic interactions, and hydrogen bond variations, this research surmises TRP86, TYR108, GLN194, TYR251, and GLU283 are the main regions of important amino acids in CCR5. In addition to the detection of TCM compound efficacy, we suggest saussureamine C is better than the others for maintaining protein composition during protein-ligand interaction, based on the structural variation.

## 1. Introduction

The human immunodeficiency virus (HIV) is a retrovirus that causes the acquired immunodeficiency syndrome (AIDS) [1–4]. In AIDS, the immune system is compromised by the virus, which then allows opportunistic infections and cancers to flourish. The HIV virus is transmitted via unprotected sexual intercourse [5, 6], contaminated medical equipment (blood transfusion, surgery, and the sharing of needles by drug addicts) [7, 8], vertical infection (pregnancy, delivery, or breastfeeding) [9, 10], and bodily fluids.

Since the first case in 1981, AIDS has caused nearly thirty-six million deaths and, in 2012 there were approximately seventy-five million carriers, as recorded by UNAIDS (http://www.unaids.org/en/resources/campaigns/globalreport2013/factsheet/). Currently, there are still no vaccines or drugs available to kill the virus and so highly active antiretroviral therapy (HAART) is the standard of care for patients with advanced infection [11]. HARRT uses a complex of transcription inhibitors to decrease the patient's total burden of HIV, but this treatment is expensive and medical costs are a social liability.

Recent research has pointed out that the C-C chemokine receptor type 5 (CCR5) is an important target for AIDS [12]. The chemokine receptor CCR5 is a receptor for the T cells that play a central role in cell-mediated immunity against viruses and pathogens. CCR5 and C-X-C chemokine receptor type 4 (CXCR4) have been defined as coreceptors for the HIV antigen gp120. HIV can infect the cell by targeting these receptors [13, 14]. Thus, preventing HIV from targeting the receptor might prevent HIV infection [15, 16]. Based on these observations, the antiretroviral drug Maraviroc has been used as it is a CCR5 receptor antagonist, thereby blocking the HIV protein from associating with the receptor.

Computer-aided drug design (CADD) is a* in silico* simulation technique to screen for novel compounds by their structure and to predict the biological activity of drug candidates. In comparison with traditional drug design, CADD has the advantages of both greater speed and lower cost. The two major application areas of CADD are structure-based drug design and ligand-based drug design. We used CADD to investigate the basics of molecular simulation in drug design centered on structure-based drug design and molecular dynamics [17–20].

Recently, there is much attention on personalized medicine and biomedicine [21, 22]; then, this knowledge could analyze the mutation [23, 24], pathway [25, 26], the cause for special disease [27–29], and the clinical diagnosis case [30]. Traditional Chinese medicine (TCM) is a model of personalized medicine. TCM has an important role in Asia, especially in China, Taiwan, Korea, and Japan. The TCM Database@Taiwan (http://tcm.cmu.edu.tw/) [31] is the largest traditional Chinese medicine database in the world. This database contains 2D chemical structures, 3D chemical structures, bioactivity, and molecular information for over 61,000 compounds of traditional Chinese medicinal herbs. The exhaustive and scientific information of traditional Chinese medicine that ZNIC could not provide could help researchers screen for multiple unadulterated TCM components. Since 2011, there have been successful discoveries of novel lead compounds from the TCM Database@Taiwan for cancer treatment [32–35], stroke prevention [36], EGFR inhibition [37], inflammation inhibition [38], pain relief [18], and antivirals [39–42]. With the assistance of the application system of the website [43] and the cloud computing platform [44], the TCM Database@Taiwan could be valuable for TCM application and drug design.

In this study, we screen a possible lead compound against HIV from the TCM Database@Taiwan. We use the computational techniques of docking simulation to select ligands. Finally, we apply molecular dynamics (MD) simulation to investigate variations from protein ligand interactions that may contribute to the evaluation of the effect of CCR5 inhibition.

## 2. Materials and Methods

### 2.1. Data Set

Accelrys Discovery Studio 2.5 (DS 2.5) was used for the molecular simulations. A total of 61,000 TCM compounds were downloaded from the TCM database (http://tcm.cmu.edu.tw/). The CCR5 (PDB ID: 4MBS) crystal structure was obtained from RCSB Protein Data Bank. Based on the literature, Maraviroc was used as a control [12].

### 2.2. Disorder Protein Detection

We take the protein structure and docking site to predict the disorder region by the database of protein disorder (DisProt: http://www.disprot.org/) because the disorder plays an important role in drug design. According to the prediction, we can decide the character of the docking site and the efficacy of the drug [45, 46].

The docking site was designed nearby the important amino acids Tyr37, Trp86, Tyr89, Trp94, Tyr108, Phe109, Phe112, Gly163, Ser179, Gln188, Gln194, Thr195, Ile198, Trp248, Tyr251, Leu255, Asn258, Thr259, Met279, Glu283, and Met287 which were defined as the active sites, V3 region, R5 region, and gp120 binding region in CCR5 [16]. The reference indicates that Maraviroc can inhibit CCR5 by targeting these regions [12].

After a comparison of the disorder regions and the docking sites, we could assess the protein-ligand interaction and drug efficacy.

### 2.3. Molecular Docking

The docking simulation used the LigandFit [47] module to dock Maraviroc and TCM compounds to CCR5 in the force field of CHARMm [48]. LigandFit is a receptor-rigid docking algorithm program in Discovery Studio 2.5 (DS 2.5). The docking site of CCR5 was identified by the research [12]. After docking, the top three docking scores of the compounds were selected and then analyzed for hydrophobic interactions by LIGPLOT [49, 50].

### 2.4. Molecular Dynamics Simulation

These ligands must be reprepared by using SwissParam (http://swissparam.ch/) [51] before applying MD simulation based on the reference force field [52] of GROMACS 4.0.7 [53]. The CCR5 protein combines with ligands as the complex goes into the buffer (or solution) simulation box. This cubic box, with a minimum distance of 1.2 Å from the complex, was solvated with the TIP3P water model in which sodium and chloride ion were added to neutralize complex charges. The complex was minimized with the steepest descent method for 5,000 steps. The last structure of minimization was transferred to MD simulation. The calculations for electrostatic interactions were based on the particle-mesh Ewald (PME) method [54]. In the PME method, each time step was 2 fs and the numbers of steps were 2,500,000 times. The equilibration under the 100 ps constant temperature (PER ensemble) was based on the Berendsen weak thermal coupling method. The total simulation time of MD was 5,000 ps. MD trajectories, RMSD, and energy variations of the complex were analyzed using a series of protocols in Gromacs. And the global MD algorithm is as follows.

*(1) Input Initial Conditions*

 Potential interaction *V* as a function of atom positions.
 
*r* and *v* are positions and velocities of all atoms in the system, respectively.

*(2) Compute Forces*. The force on any atom defined as (1)

(1) $$\begin{array}{c} F_{i}=-\frac{\partial V}{\partial r_{i}} \end{array}$$

is computed by calculating the force between nonbonded atom pairs:

(2) $$\begin{array}{c} F_{i}=\sum_{j}F_{ij} \end{array}$$

the forces was added due to bonded interactions (which may depend on 1, 2, 3, or 4 atoms) plus restraining and/or external forces. At the same time, the potential and kinetic energies and the pressure tensor are computed.

*(3) Update Configuration*. The movement of the atoms is simulated by numerically solving Newton's equations of the atom motion:

(3) $$\begin{array}{c} \frac{d^{2}r_{i}}{{dt}^{2}}=\frac{F_{i}}{m_{i}}. \end{array}$$

*(4) Output Step*. Write positions, velocities, energies, temperature, pressure, and so forth.

Then, repeat (2), (3), (4) for the required number of steps.

## 3. Results and Discussion

### 3.1. The Detection of Disorder Protein

**Figure 1:**
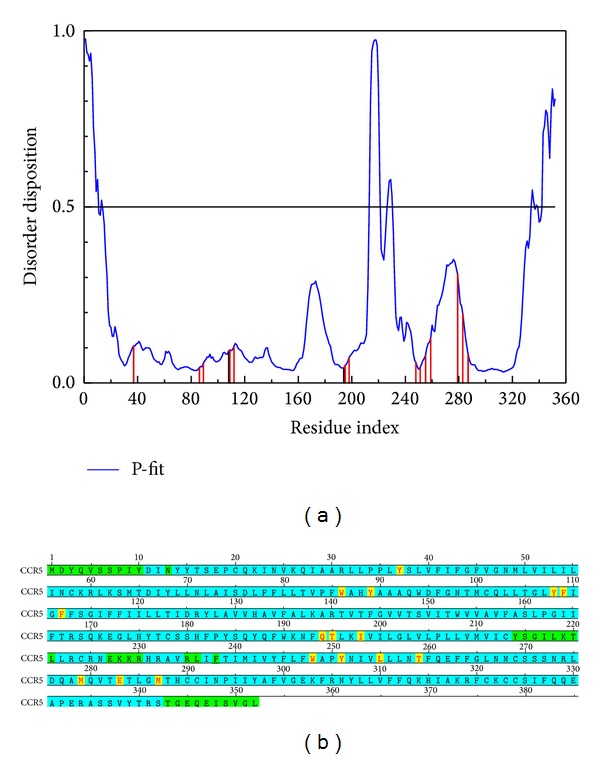
The disorder and binding site detection. The blue curve in the top figure is the disorder disposition of each amino acid, and the red lines are the residues of the important amino acids. The following diagram describes the information at the top. The green regions in the amino sequence indicate the predicted disorder regions and the yellow regions with red lettering identify the important amino acids.

The disorder protein is intrinsically an unstructured protein. For this character, while the docking site consists of a disorder region, the drug dock to protiein and the complex will stablize difficultly. But our cited references also indicate that the disorder region is not any defined domain; therefore, the drug can interact with disorder region which may have lower side effect than interacting with the domain which is widespread in body. For above discussion, the disorder region can be defined as a hard work for drug design better than as a bad docking site for selection. The disorder regions of CCR5 are defined as having a disposition of over than 0.5 (Figure 1). This result indicates that the important amino acids do not consist of disorder regions; thus, the ligand docks to the selected site which is appropriate and our results have a weaker effect than disorder protein. For above reasons, the compounds selected based on docking could have an influence on CCR5.

### 3.2. Molecular Docking

After molecular docking and ranking by docking score, the top three TCM compounds can be selected (Table 1). These TCM compounds are saussureamine C, 5-hydroxy-L-tryptophan, and abrine derived from the TCM herbs* Saussurea lappa* Clarke,* Mucuna pruriens* (L) D.,* and Abrus precatorius* L. (or* Abrus fruticulosus* Wall. ex Wight et Arn.), respectively. The top compound, saussureamine C, is defined as an antiulcer compound [55] and the herb* Saussurea lappa* Clarke can prevent breast cancer cell migration [56] and treat heart disease [57, 58], has antihepatotoxic activity [59], and inhibits the killing function of cytotoxic T lymphocytes [60]. The second ranked herb,* Mucuna pruriens*, can reduce oxidation and prevent Parkinson's disease [61, 62]. The third ranked compound, abrine from the herb* Abrus precatorius*, has an immune-toxin [63] and can induce apoptosis [64, 65]. As reported in the literature, most of these compounds can have an effect on immunity. For the above reasons, we suggest that the selected compounds can have an influence on T-cell receptors, such as CCR5.

**Figure 2:**
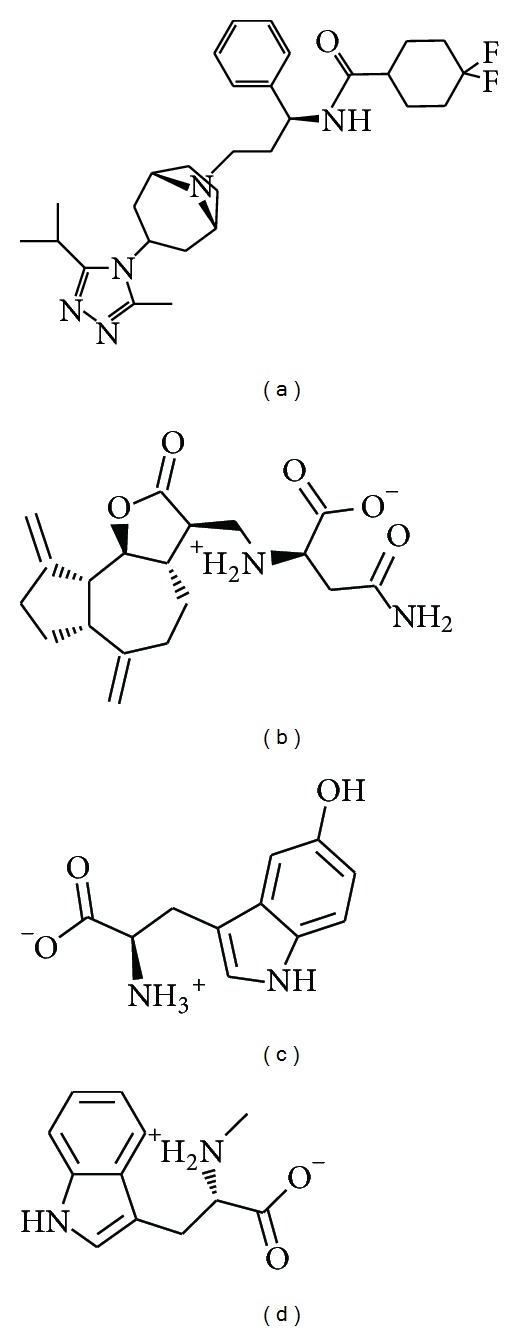
The structure of control and candidate TCM compounds. (a) Maraviroc, (b) Saussureamine C, (c) 5-hydroxy-L-tryptophan, and (d) abrine.

**Figure 3:**
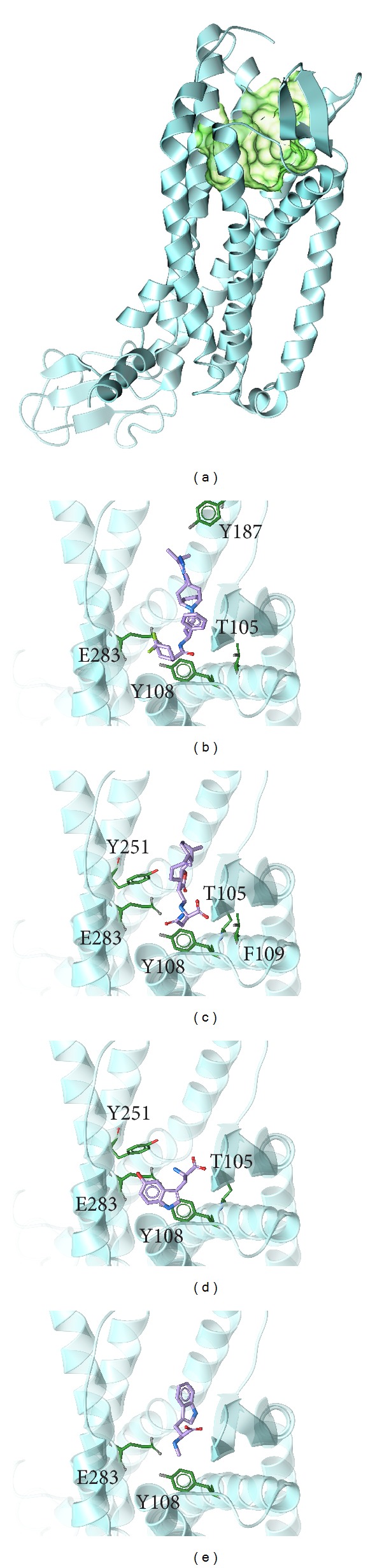
The docking poses of ligands. (a) The crystal structure of CCR5 and the docking site, (b) Maraviroc, (c) Saussureamine C, (d) 5-hydroxy-L-tryptophan, and (e) abrine.

The structure of the candidate compounds and control were selected after screening first the TCM database (Figure 2), then the docking poses sign, the docking site, and the amino acid neighbors by ligands (Figure 3). From this result, we observe Tyr108 and Glu283 are defined as the amino acids that can interact with all ligands (the control and selected compounds); thus, these two amino acids may play important roles in a CCR5 target function.

**Figure 4:**
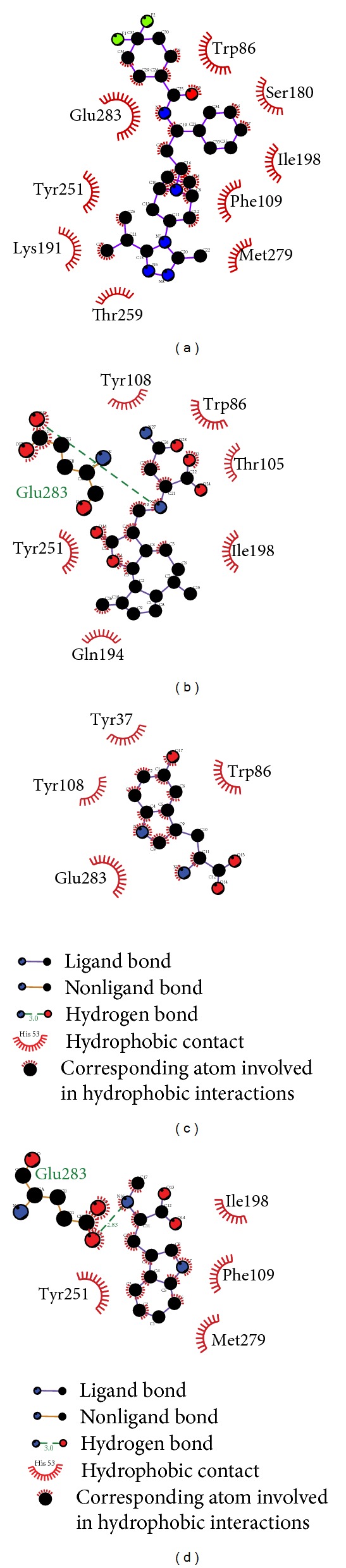
LIGPLOT illustrates the protein-ligand interactions. (a) Maraviroc, (b) Saussureamine C, (c) 5-hydroxy-L-tryptophan, and (d) abrine. The deep red color of the hydrophobic interactions indicates a high frequency in all ligand interactions.

The hydrophobic interaction can be analyzed by LIGPLOT (Figure 4). This result shows that the amino acids Trp86, Phe109, Ile198, Tyr251, Met279, and Glu283, colored deep red, are at a high frequency, while proteins have interactions with the ligands through hydrophobic interactions or hydrogen bonds. These amino acids have been defined as important amino acids in the literature,; thus, the hydrophobic interaction analysis is credible and the selected compounds can have an effect on CCR5.

### 3.3. Molecular Dynamics Simulation

We calculated the H-bond distance to decide the H-bond occupancy during MD (Table 2). In Table 2, we find that the defined important amino acids, Trp86, Tyr108, Gln194, Ser179, Tyr251, and Glu283, will have an H-bond, even if the greater H-bond occupancies of abrine are not important amino acids. This phenomenon may show that the above amino acids are not only important for target functions, but are major points of immunity regulation. Abrine may cause some functional differences from the others by subordinate amino acids.

**Figure 5:**
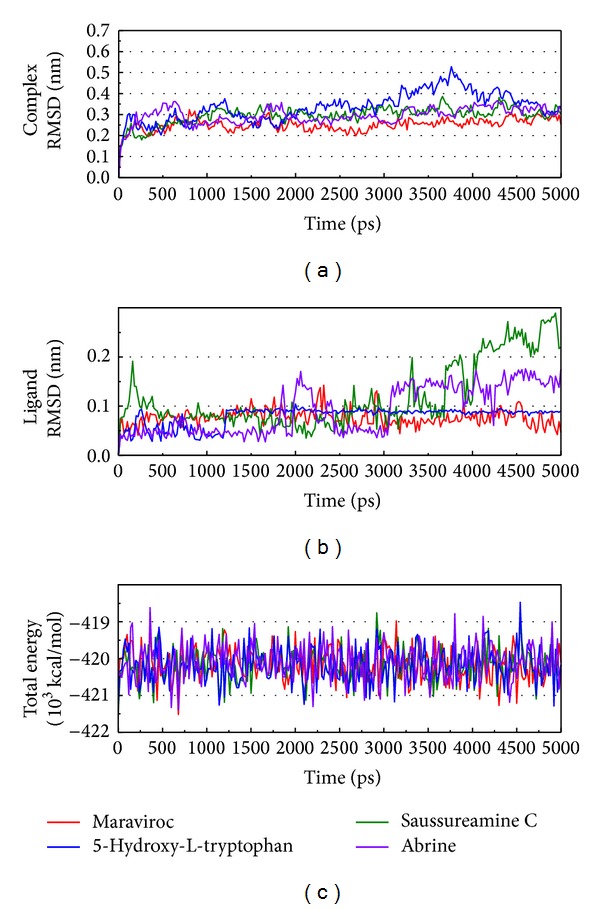
Measures of the MD trajectories. (a) Complex RMSD, (b) ligand RMSD, and (c) the total energy.

The RMSD and total energy of a complex during MD simulation were recorded (Figure 5). The total energy is in the range of −1755 ~ −1745∗10^3^ kcal/mol and tends to −1750∗10^3^ kcal/mol. The amplitude indicates the complex is still interacting. The compounds saussureamine C and abrine have a high variation in ligand RMSD; the structure of these two compounds is twisted during the interaction. The complex RMSD tends to 0.3 nm and the curve becomes gentle; thus, this result indicates the complex is stable and the interaction will be balanced.

From the above, we determine that the protein and ligand can interact, which leads us to discuss the ligand-effect based on the structure variation from the beginning of the MD to completion.

**Figure 6:**
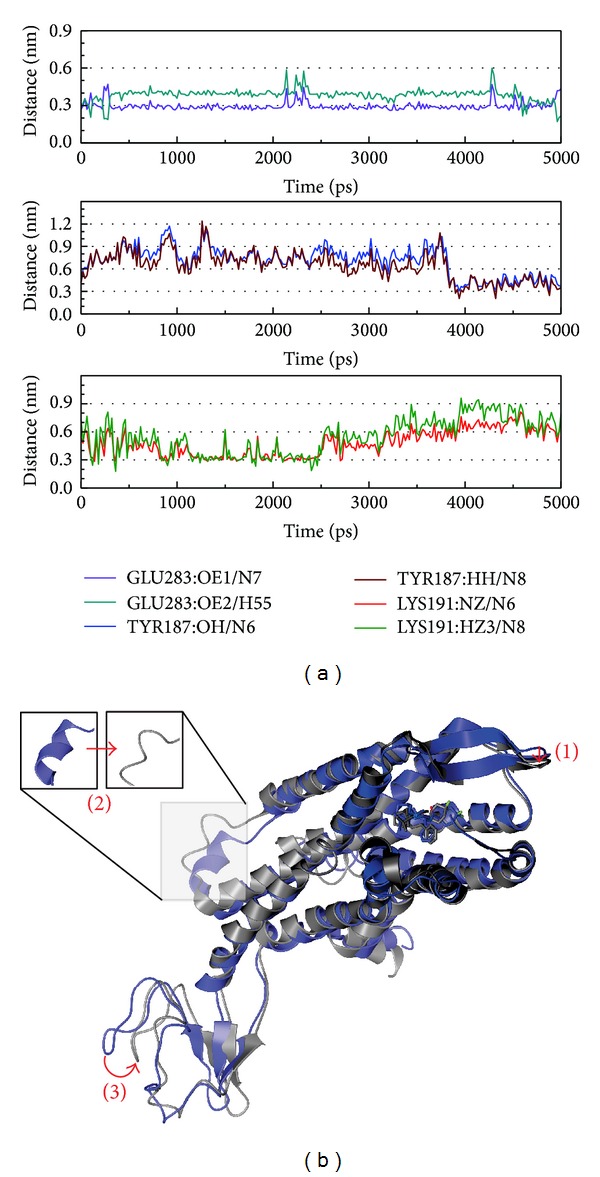
The variation of Maraviroc and CCR5 complex in MD simulation. (a) H-bond variation and (b) structure variation. The (1)–(3) red color indicates the difference through MD.

The variation in H-bonds and the Maraviroc-CCR5 complex are recorded (Figure 6). In Figure 6(a), the H-bond is below 3 nm (indicating H-bond production is possible). It was found that the H-bond that consisted of Lys191 was in prophase, Tyr187 was in late stage, and Glu283 was full-time. We suggest that Lys191 may help the target, Tyr187 has effect on immunity, and Glu283 is the main point for CCR5 in the control case. In Figure 6(b), CCR5 has three differences after MD simulation. The CCR5 is a transmembrane protein and according to the variation, we suggest the difference (1) indicates the target site is close, while the complex interacts to prevent other drugs or peptides snatching the docking site. Difference (2) changes the structure (the helix becomes a loop or other), and difference (3) indicates a change in position may cause the divergence function in the inner membrane. Based on the above discussion, this experience can be observed and examined in other compounds.

**Figure 7:**
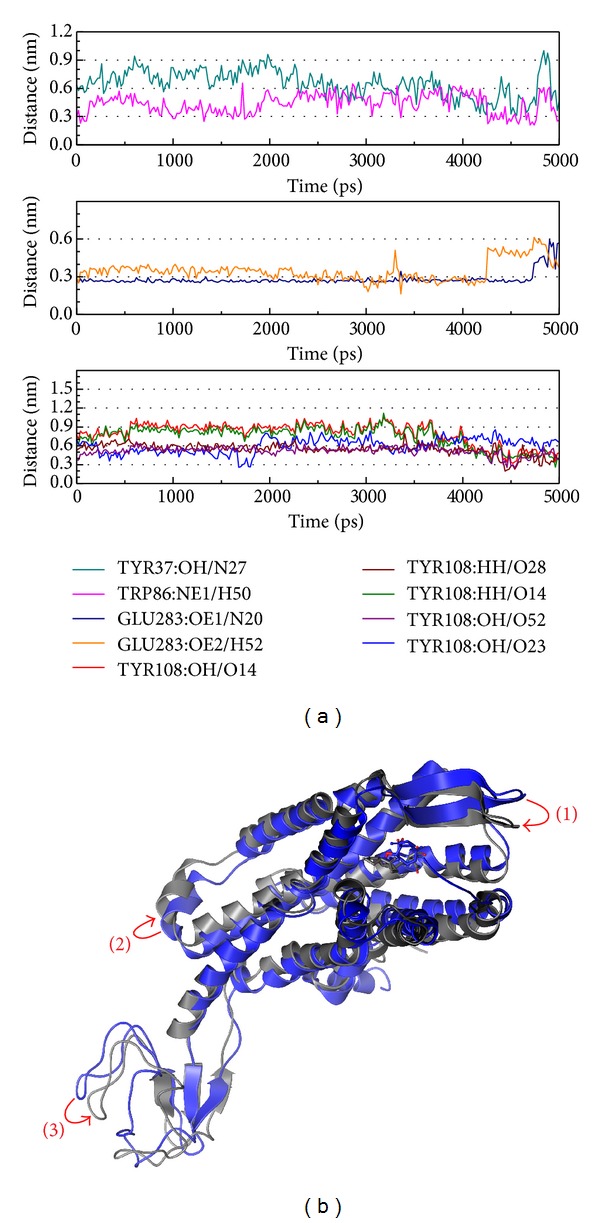
The variation of saussureamine C and CCR5 complex in MD simulation. (a) H-bond variation and (b) structure variation. The (1)–(3) red color indicates the difference through MD.

The complex of saussureamine C and CCR5 are analyzed (Figure 7). In Figure 7(a), the data shows Tyr108 is in a late stage function and Trp86 is a full-time effect, but weaker than Glu283. In Figure 7(b), the structural variation is different from the control. The complex of saussureamine C and CCR5 only changes the position and has a larger variation than in the control. This condition may be better than the control in that the saussureamine C does not cause the structure composition to break and thus makes the protein conformation maintain the complex interaction.

**Figure 8:**
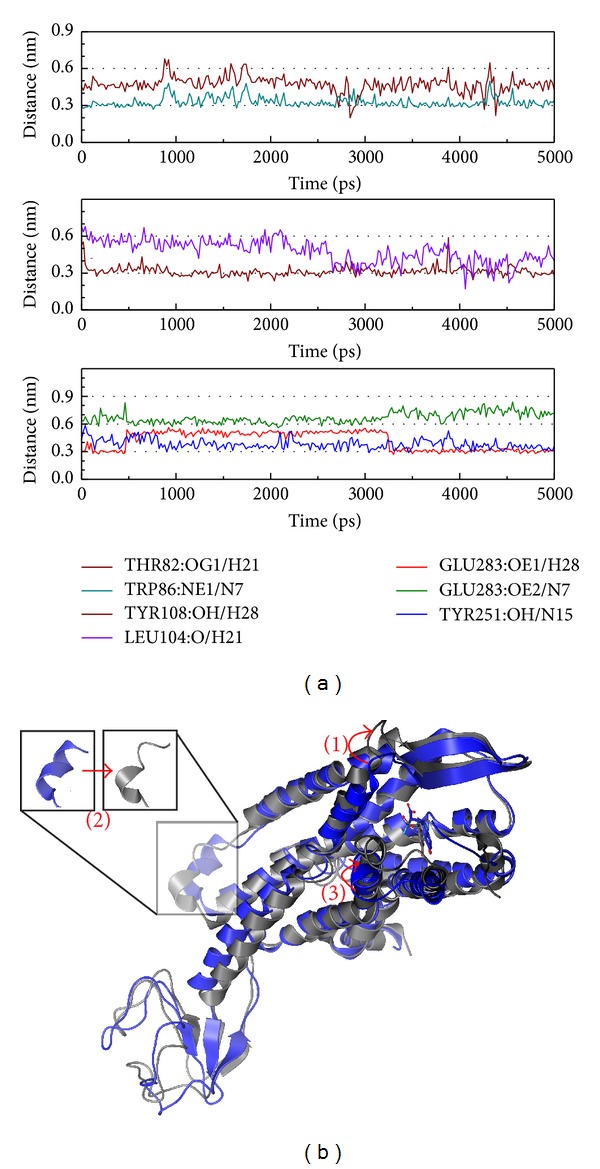
The variation of 5-hydroxy-L-tryptophan and CCR5 complex in MD simulation. (a) H-bond variation and (b) structure variation. The (1)–(3) red color indicates the difference through MD.

The 5-hydroxy-L-tryptophan effects on CCR5 are presented in Figure 8. In this result, 5-hydroxy-L-tryptophan makes Trp86, Tyr108, and Tyr251 express a function stronger than Glu283. In Figure 8(b), the main structural variation of the complex is in the outside membrane close to the target site and is similar to the control in that the structural composition is broken in the inner membrane. From these results, we can surmise that 5-hydroxy-L-tryptophan may have a greater effect on preventing other drug noise, rather than cell reaction.

**Figure 9:**
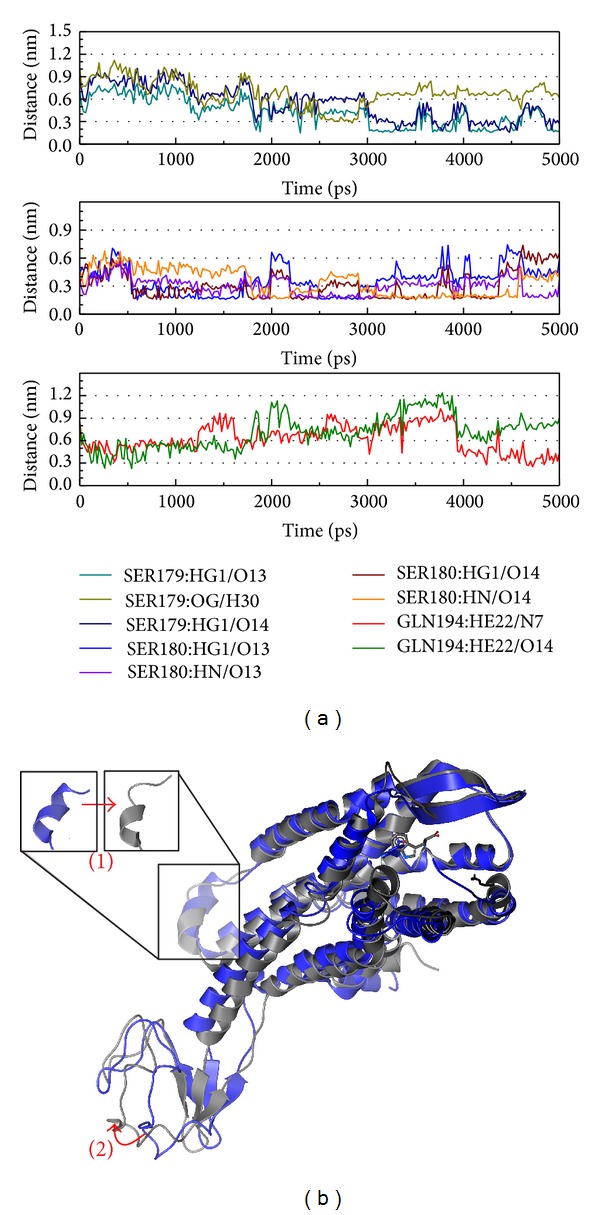
The variation of abrine and CCR5 complex in MD simulation. (a) H-bond variation and (b) structure variation. The (1)-(2) red color indicates the difference through MD.

The abrine complex interactions were recorded (Figure 9). In Figure 9(a), besides Ser179 that consisted of a late stage function, Ser180 becomes the main expression and is defined as being important. Thus, abrine may have a different function from the other compounds. Although the structural composition is broken, similar to 5-hydroxy-L-tryptophan, abrine only causes a variation in the inner membrane. From this difference, we suggest abrine may have an influence on immunity but may be weak in drug competition.

## 4. Conclusion

Based on above discussion, we find the top three TCM compounds saussureamine C, 5-hydroxy-L-tryptophan, and abrine can have an effect on CCR5 against HIV infection. Trp86, Tyr108, Gln194, Ser179, Tyr251, and Glu283 present their effects on CCR5 through hydrophobic interactions and H-bonds, especially Glu283, which can be verified as a key residue in CCR5. The structural variations indicate all compounds can have an effect on immunity function, but saussureamine C is better than the other compounds as it can maintain the protein composition.

## Figures and Tables

**Table 1:** Scoring functions of the top three compounds and the inhibitors of CCR5.

Compounds	Herbs	-PLP1	-PLP2	Dock score
Saussureamine C	*Saussurea lappa *Clarke	60.11	57.39	201.096
5-Hydroxy-L-tryptophan	*Mucuna pruriens *	44.4	43	181.038
Abrine	*Abrus precatorius *L. *Abrus fruticulosus *Wall. ex Wight et Arn.	26.78	23.39	170.166
Maraviroc*		73.2	71.58	67.369

*: control.

**Table 2:** H-bond occupancy for CCR5 (4MBS) with Maraviroc and the top three TCM compounds.

Name	H-bond interaction	Occupancy
Maraviroc	TYR187:HH/N8	2%
	LYS191:NZ/N6	6%
	LYS191:HZ3/N8	6%
	GLU283:OE1/N7	73%
	GLU283:OE2/H55	4%

Saussureamine C	TRP86:NE1/H50	13%
	TYR108:OH/O23	2%
	TYR108:HH/O28	4%
	TYR108:OH/H52	2%
	GLU283:OE1/N20	94%
	GLU283:OE2/H52	28%

5-Hydroxy-L-tryptophan	THR82:OG1/H21	3%
	TRP86:NE1/N7	24%
	LEU104:O/H21	4%
	TYR108:OH/H28	33%
	TYR251:OH/N15	2%
	GLU283:OE1/H28	12%

Abrine	GLN194:HE22/N7	2%
	GLN194:HE22/O14	2%
	SER179:HG1/O13	30%
	SER179:HG1/O14	21%
	SER179:OG/H30	1%
	SER180:HG1/O13	36%
	SER180:HG1/O14	58%
	SER180:HN/O13	44%
	SER180:HN/O14	47%

H-bond occupancy cutoff: 3.0 Å.
